# Supplementary figures and images for: Socioeconomic inequalities associated with Geriatric syndrome in Thailand: The results of Fifth National Health Examination Survey
Source: PLoS One. 2024 Oct 10;19(10):e0311687. doi: 10.1371/journal.pone.0311687 (PMC11469603; doi:10.1371/journal.pone.0311687)

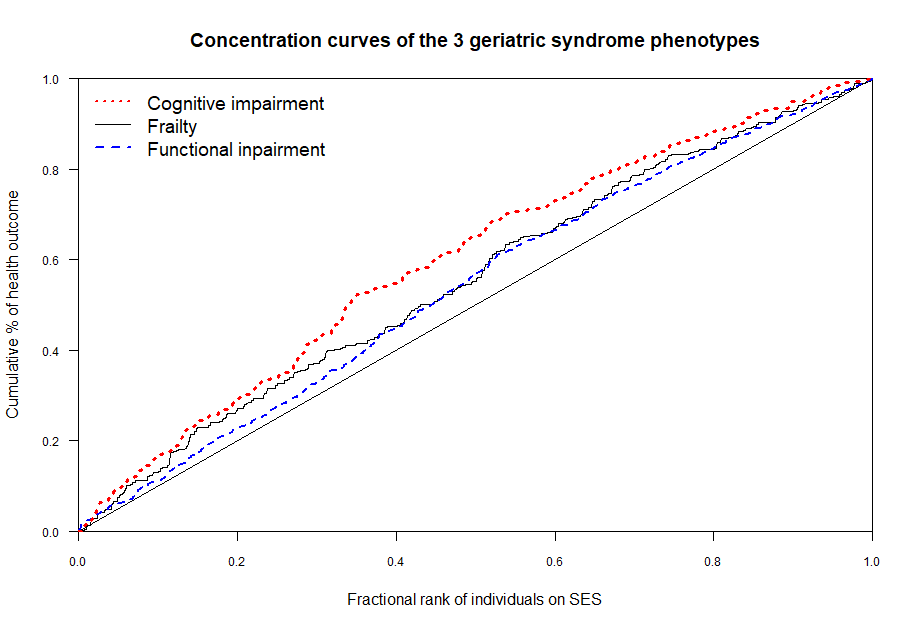

Supplement: S1 Fig — (TIF) [file pone.0311687.s001.tif]
