# Supplementary material for: Socioeconomic inequalities associated with Geriatric syndrome in Thailand: The results of Fifth National Health Examination Survey
Source: PLoS One. 2024 Oct 10;19(10):e0311687. doi: 10.1371/journal.pone.0311687 (PMC11469603; doi:10.1371/journal.pone.0311687)
Supplement: S1 File — (PDF) [file pone.0311687.s002.pdf]

Table A: Summary statistics of socioeconomic and sociodemographic variables related with the decomposition analyses

Variable	Weighted %	C	C_n	$ß^m$ for NCD	$ß^m$ for FI	$ß^m$ for Frailty
**Age (ref = 60 - 69 year)**						
70 - 79 years	0.300	-0.019	-0.027	0.026	0.067	0.051
80 years or more	0.131	-0.100	-0.116	0.140	0.126	0.115
**Gender (ref = female)**						
Male	0.454	0.011	0.020	-0.010	-0.058	0.002
**Marital status (ref = married)**						
Divorce/ separated	0.064	0.003	0.003	-0.022	0.015	0.006
Widowed	0.270	-0.046	-0.063	0.014	0.018	0.002
Single	0.057	-0.067	-0.071	0.059	-0.027	-0.009
**Education (ref = primary school or lower)**						
High school or higher	0.123	0.473	0.540	0.035	-0.025	-0.013
**Working status (ref = unemployed)**						
Employed	0.404	-0.020	-0.033	-0.041	-0.031	-0.049
**UCS (ref = no)**						
Yes	0.853	-0.061	-0.419	0.025	-0.033	0.007
**SSS (ref = no)**						
Yes	0.033	0.301	0.311	-0.028	0.077	-0.018
**CSMBS (ref = no)**						
Yes	0.057	0.511	0.542	0.019	-0.022	0.009
**Other (ref = no)**						

Yes	0.104	0.170	0.190	-0.031	-0.046	-0.001
**SES tercile (ref = tercile 1)**						
Tercile 2	0.333	0.004	0.006	-0.023	0.018	-0.003
Tercile 3	0.331	0.669	1.000	-0.048	-0.032	-0.024
**Region (ref = Bangkok)**						
North	0.247	-0.054	-0.071	0.020	0.029	-0.003
Central and West	0.231	0.068	0.088	-0.015	-0.045	-0.005
Northeast	0.349	-0.196	-0.300	0.004	0.055	-0.028
South	0.067	0.044	0.047	0.030	0.100	-0.016

Remarks: C Concentration index; C_n normalized concentration index; $ß^m$ Beta coefficients (marginal effects evaluated at the parameter mean); UCS Universal Health Coverage; SSS Social Security Scheme; CSMBS Civil Servant Medical Benefit Scheme; NCD Neurocognitive Disorder (Cognitive impairment); FI Functional impairment

Table B: Normalized concentration index and contribution of socioeconomic and sociodemographic determinants to cognitive impairment, functional dependency, and frailty

	**Cognitive impairment**		**Functional impairment**		**Frailty**	
C	-0.282		-0.275		-0.181	
C^{is}	-0.254		-0.261		-0.150	
	**AC**	**%CC**	**AC**	**%CC**	**AC**	**%CC**
**Need variables**						
Age	-0.027	13.8	-0.013	16.2	-0.031	28.1
Gender	-0.001	0.5	-0.003	3.5	<0.001	-0.2
**Subtotal**	**-0.028**	**14.3**	**-0.016**	**19.7**	**-0.031**	**27.9**
**Non-need variables**						
Marital status	-0.006	2.8	-0.001	1.3	0.001	-0.1
Education	0.027	-13.9	-0.008	11.0	-0.013	11.5
Working status	0.006	-3.3	0.002	-2.8	0.01	-8.5
UCS	-0.104	53.2	0.058	-77.1	-0.039	34.4
SSS	-0.003	1.7	0.004	-5.3	-0.003	2.4
CSMBS	0.007	-3.5	-0.003	4.5	0.004	-3.8
Other insurance	-0.007	3.6	-0.005	6.0	<0.001	0.2
SES	-0.185	94.2	-0.053	69.7	-0.114	101.6
Region	-0.011	6.0	-0.035	45.3	0.042	-36.7
**Subtotal**	**-0.276**	**140.8**	**-0.041**	**52.7**	**-0.112**	**101.0**
Unexplained C	0.094		0.197		-0.009	

Remarks: C, concentration index; C^{is} indirectly standardized concentration index; AC, Absolute contribution to the concentration index; %CC, Percentage contribution to the concentration index.
