# Supplementary material for: Socioeconomic inequalities associated with Geriatric syndrome in Thailand: The results of Fifth National Health Examination Survey
Source: PLoS One. 2024 Oct 10;19(10):e0311687. doi: 10.1371/journal.pone.0311687 (PMC11469603; doi:10.1371/journal.pone.0311687)
Supplement: Attachment — Submitted filename: Response to Reviewers.docx [file pone.0311687.s003.docx]

Dear Editor,

We thank you for the opportunity to revise our manuscripts and appreciate the work of the referees and the comments from the reviewers. We carefully reviewed the comments and provided our response for each comment.

**Responses to comments regarding the journal requirements**

| N | Comment | Response |
| --- | --- | --- |
| 1. | When submitting your revision, we need you to address these additional requirements.  Please ensure that your manuscript meets PLOS ONE's style requirements, including those for file naming. The PLOS ONE style templates can be found at  https://journals.plos.org/plosone/s/file?id=wjVg/PLOSOne_formatting_sample_main_body.pdf and  https://journals.plos.org/plosone/s/file?id=ba62/PLOSOne_formatting_sample_title_authors_affiliations.pdf | We revised the manuscript and prepared it per the journal guideline and also consulted Editage editing service to make sure formats of the manuscript and its supplementary files are in line with the journal requirements. |
| 2. | We note that the grant information you provided in the ‘Funding Information’ and ‘Financial Disclosure’ sections do not match.  When you resubmit, please ensure that you provide the correct grant numbers for the awards you received for your study in the ‘Funding Information’ section. | We have provided grant information in the ‘Funding Information’ section. We did not indicate the name of authors who received funding support because we analyzed de-identified data of the fifth National Health Examination Survey (NHES-5) with the permission from the NHES committee; and NHES-5 itself was supported by the funding sources: Bureau of Policy and Strategy, Ministry of Public Health (grant number 57–054), Thai Health Promotion Foundation (grant number 55-00-1177), and the National Health Security Office, Thailand (no grant number). |
| 3. | We note that you have indicated that data from this study are available upon request. PLOS only allows data to be available upon request if there are legal or ethical restrictions on sharing data publicly. For more information on unacceptable data access restrictions, please see http://journals.plos.org/plosone/s/data-availability#loc-unacceptable-data-access-restrictions.  In your revised cover letter, please address the following prompts:  a) If there are ethical or legal restrictions on sharing a de-identified data set, please explain them in detail (e.g., data contain potentially sensitive information, data are owned by a third-party organization, etc.) and who has imposed them (e.g., an ethics committee). Please also provide contact information for a data access committee, ethics committee, or other institutional body to which data requests may be sent.  b) If there are no restrictions, please upload the minimal anonymized data set necessary to replicate your study findings as either Supporting Information files or to a stable, public repository and provide us with the relevant URLs, DOIs, or accession numbers. For a list of acceptable repositories, please see http://journals.plos.org/plosone/s/data-availability#loc-recommended-repositories.  We will update your Data Availability statement on your behalf to reflect the information you provide | We added information about the restrictions in data availability in our covering letter.  “However, we cannot make publicly available the data we used for the current analysis as the investigator committee of the Fifth National Health Examination Survey owns it. The restriction applied to data sharing could be lifted and the data could be made available upon reasonable request (Please contact: Professor Wichai Aekplakorn, Department of Community Medicine, Faculty of Medicine, Ramathibodi Hospital, Bangkok, Thailand: wichai.aek@mahidol.ac.th).” |
| 4. | Please include captions for your Supporting Information files at the end of your manuscript, and update any in-text citations to match accordingly. Please see our Supporting Information guidelines for more information: <http://journals.plos.org/plosone/s/supporting-information>. | We added captions for our supporting information at the end of the manuscript and made sure all in-text citations are in line with the details. We also used Preflight Analysis and Conversion Engine (PACE) digital diagnostic tool to make the figure format consistent with the requirements. |
| 5. | Please review your reference list to ensure that it is complete and correct. If you have cited papers that have been retracted, please include the rationale for doing so in the manuscript text, or remove these references and replace them with relevant current references. Any changes to the reference list should be mentioned in the rebuttal letter that accompanies your revised manuscript. If you need to cite a retracted article, indicate the article’s retracted status in the References list and also include a citation and full reference for the retraction notice. | We have reviewed the reference list and ensured that the references are relevant to the recent manuscript version. |

**Response to reviewer’s comments**

Reviewer #1:

| N | Comment | Response |
| --- | --- | --- |
| 1. | Abstract:  - The authors propose some applied suggestions for reducing inequality.  - Please provide numerical values for concentration index and decomposition. | We added numerical values of indirectly standardized concentration index and percentage of contribution to the concentration index in the relevant sections.  Page 2 line 38  “Contributions to the concentration index (CC)—contribution to a more or less equitable GS distribution—were decomposed and shown in terms of percentage and direction All GS phenotypes were found to be concentrated in the elderly poor (C*^is^* of FI, frailty, and NCD = - 0.068, -0.092, and -0.182, respectively). Work status contributes to a more equitable GS distribution in all the phenotypes (%CC in FI, frailty, and NCD = - 1.7%, -5.1%, and -2.0%, respectively), whereas types of insurance schemes made bidirectional contributions to the equity of GS.” |
| 2. | Background:  - It is need to be explained what are the gaps in the literature that authors wanted to address before reporting this research- in other words, what is already known and what does this article add to the literature? | We emphasize the gap of knowledge in the latter part of our introduction by statements as following.  Page 4 line 96  “Equitable implementation of policies and interventions in GS requires information on the SES of the target population, but such information is inconsistent in Thailand. Earlier studies have shown the degree of socioeconomic inequality regarding aging and the general health of older individuals for past decades; however, there is still a lack of information about inequality in GS distribution. Further, priority setting for capacity building and policy development requires more in-depth information on the extent each socioeconomic determinant contributes to inequality.” |
| 3. | Results:  Table 1 must be reported in the separate page. | We modified the descriptions pertaining Table 1 information to elaborate details relevant to the discussions and reported the Table in a separate page. The lower bound of the region estimation (Bangkok) was also changed from -9.1% to 0.0% as a legitimate value for the parameter’s bound (percentage).  Page 10 line 233  “Approximately 40% of participants worked actively, while the remaining majority were either unemployed or housemakers. More than 4,000 individuals had UCS (85.3%), whereas 220, 414, and 610 individuals (3.3%, 5.7%, and 10.0%) had SSS, CSMBS, and other health insurance plans (e.g., private insurances and state enterprise schemes), respectively. Six hundred and forty-five individuals reported that they had used two or more health insurance plans. The southern region was the least common area of residence of the study participants (6.7%). The participants’ SES were relatively balanced among the three levels.” |
| 4. | Discussion:  Discussion should focus on critical interpretation of the findings. The study finding must be compared with existing agreed and disagreed cases. In this study, I don't see any comparison with disagreed cases. | We added discussion points to compare the current findings with some disagreed cases regarding the impact of health schemes to elderly health and discuss them with the current findings.  Page 17 line 330  “The current study showed that active work contributed to a more equitable distribution of GS in the older population. The multifactorial model explained GS by interactive accumulated biological risks across an individual’s lifetime and linkage with psychosocial factors.^5^ Further, some studies found that employment could reduce the risk of cognitive impairment in later life.^46,47^ We speculate that the narrowing gap in GS between well-off and poor individuals could be caused by the presence of working activities. However, a previous study found that only time away from work caused by early unemployment, sickness, and voluntarily homemaking increased the risk of NCD in older individuals while work leave for training and maternity might reduce the risk of NCD.^47^ Thus, caveats should be considered that promoting active-working status at some points before the retirement age might reduce equity gaps only in some cases of unemployed individuals.”  And in another paragraph,  Page 18 line 365  “A previous study conducted among the Chinese elderly^24^ emphasized that disparities and fragmentations of the health insurance scheme significantly impacted the overall self-rated health of the elderly whereas another study in rural China^57^ found no significant correlation between health and health insurance. We argue that the strategy to deal with these bidirectional contributions and overall elderly health regarding GS, which enables individuals’ access to a certain health scheme (resulting in either more or less equitable distributions in certain GS phenotypes) might not be as effective as enhancing equality of health scheme access between the rich and the poor (making inequalities converge towards zero).”  Some statements were also modified to clarify the direction of association between risk factors and geriatric syndrome as follows:  Page 16 line 319  “Therefore, several studies have investigated the possible determinants of these phenotypes of GS. According to previous studies, multiple socioeconomic and sociodemographic factors, including being females, having lower education,^20,44,45^ having a lower income,^20,45^ and unemployment,^20^ contributed to the increased likelihood of NCD, FI, and frailty in older individuals.” |
| 5. | The conclusion section is pretty short. Maybe that could be extended | We extended our conclusion to give a more detailed summary as well as suggestions for policy makers and future studies.  Page 19 line 392  “Cognitive impairment, functional decline, and frailty were significantly more prevalent in the poor elderly group, of which the observed inequality was relatively larger among those with cognitive impairment. Employment status and health insurance schemes can be treated as potentially modifiable non-need factors that require attention from health policymakers. Distributions of employment status provided uniform explanation for the widening inequality of all GS phenotypes. Therefore, based on this finding, the study emphasizes the importance of working opportunity in modifying inequitable GS in old age. Bidirectional contributions from inequitable health insurance schemes suggest that equalizing access or consolidating health insurance schemes could be a more promising strategy for the country’s universal health coverage than providing access to any one of the schemes. Inequitable cognitive impairment, employment, and access to insurance schemes required further attention from policymakers to establish a healthy aging society. Nevertheless, future study should evaluate the impact of these socioeconomic contributions overtime and investigate the effectiveness of public health interventions which have been implemented.” |

Reviewer #2:

| N | Comment | Response |
| --- | --- | --- |
| 1. | 1. The title and the introduction of the manuscript sound the social science but the methodology showed the high level of statistical idea. It will be better to explain the idea of index and the statistical methods that show clearer idea of GS and inequlity. | We thank you for acknowledging the complexity of the concentration index in showing the inequality for audience in general. Thus, we added a clarification into our detailed explanations in our ‘Inequality measurements’ subsection of the method section.  Page 8 line 177  “The index typically ranges from -1 to 1, with a positive value indicating a concentration of the outcome among groups with higher SES (the outcome accumulating more among well-off individuals); a negative value suggesting that individuals in the lower SES group are more likely to experience the outcome than the affluent; and a value of zero indicating no socioeconomic-related inequality.” |
| 2. | Table 2 presented the symbol "*" to illustrate the level of p-value significant. I recommend to present with p-value. | We have changed the symbols to p-values in Table2. |
| 3. | For Table 3 and the text explanation, this section was important for answer the research question. However, it hard to understand for less knowledge of statistics. It will be better to explain more for application of this results. | We thank you for giving us the notice and see this one important for readers in general. Thus, we added a sentence explaining the meaning of the decomposition analysis, and added one point in the discussion to indicate that policy makers could also use these decompositions to prioritize the policy to manage these relevant factors.  Page 13 Line 277  “Our decomposition analysis produced AC and %CC which represent the extent of inequality in each variable that was responsible for each GS phenotype.” |
| 4. | For the limitation, from "Thus, the current findings may not represent all GS phenotypes..." What types of phenotypes that we can generalized. | We assessed that our results represent situations of inequality only in the scope of problems: neurocognitive dysfunction, functional impairment, and frailty. Thus, we added a statement to clarify the limitation in generalizability for our study results.  Page 19 line 387  “Thus, the current findings illustrate situations of inequality only in NCD, FI, and frailty, and might not be generalized to all GS phenotypes, such as decubitus ulcers and polypharmacy.” |
