# Supplementary material for: Socioeconomic inequalities associated with Geriatric syndrome in Thailand: The results of Fifth National Health Examination Survey
Source: PLoS One. 2024 Oct 10;19(10):e0311687. doi: 10.1371/journal.pone.0311687 (PMC11469603; doi:10.1371/journal.pone.0311687)
Supplement: Attachment — Submitted filename: Response to Reviewers.docx [file pone.0311687.s004.docx]

**General response**

First, we would like to express our thanks for your thorough editing and valuable suggestions for improving the manuscript. We have carefully reviewed and addressed each of your comments point-by-point.

**Point-by-point response to the editor’s comments**

• Generally, research reports are often differentiated from research articles. Hence, authors are advised to take cognizance of that. As it is, the manuscript looks more like research report instead of research article.

**Response**: We acknowledge the merits of the study and would like to thank you for your constructive comments.

• Age of participants (60+ years) should be indicated in Abstract, as mentioned in Methodology “total of 7,365 participants aged 60 years old or older were…”. That is, “Nationally representative data of 7,365 individuals aged 60 years AND ABOVE from the 5th National Health…”.

**Response**: We thank you for highlighting the need for clarifications, and we have revised the relevant text per your suggestions.
Revisions have been made on page 2, line 31

• Exclusion of participants with missing wealth index data from the NHES-5 should be clearly stated to avoid confusion. Suggestively, in Abstract, “The wealth index was used as the main indicator of socioeconomic status, and PARTICIPANTS with missing wealth index data were excluded.” Also in methodology, “Those (1904 PARTICIPANTS IN TOTAL) who did not report household assets (i.e., missing wealth index values) were excluded from the analysis”.

**Response**: We thank you for highlighting the need for clarifications, and we have revised the relevant text per your suggestions.
Revisions have been made on page 2 line 35 and page 5 line 120

• Correct minor grammatical errors such as “Therefore, BASED on this finding, the study emphasizes the importance.” Also, “Inequitable cognitive impairment, employment, and access to insurance schemes REQUIRED further attention from policymakers”. Furthermore, “Our findings that the socioeconomic inequalities of NCD, FI, and frailty in the Thai population were also explained by age, education, and working status, while age was considered an actual biological determinant of GS, and that education and employment can be treated as potentially unfairly distributed factors contribute to the literature.”

**Response**: Thank you for highlighting the need for revisions. We have updated the relevant text according to your suggestions

Revisions have been made on:

Page 17 line 326-330 (the whole sentence is rearranged)

Page 20 line 399 (“based on this finding,” has been removed)

• “were living with their couples” should be, ‘were living with their SPOUSES’.

**Response**: We thank you for highlighting the need for revisions, and we have revised the relevant text per your suggestions.

Revisions have been made on page 10 line 233

• "…not only with the increased likelihood of poorer health outcomes and mortality in the elderly,35–40 but with an increased… NOT ONLY…, BUT ALSO…

**Response**: Thank you for highlighting the need for revisions. We have updated the relevant text according to your suggestions

Revisions have been made on page 16 line 320

• Earlier reviewers noted the need to explain gap in the literature. However, authors addressed this without making reference to previous studies. For instance, “Earlier studies have shown the degree of socioeconomic inequality regarding aging and the general health of older individuals for past decades…” (REQUIRE CITATIONS)

**Response**: We thank you for your comment. we have revised our statement to more precise (using “in the last decade” instead of “for the past decades”) and added the relevant reference.

Revisions have been made on page 5 line 99

• “Age and work status were significant factors associated with GS, with a higher prevalence among older age groups” (, WIDOWED AND UNEMPLOYED).

**Response**: Thank you for highlighting the key points to emphasize in the results section. We have revised the relevant text in accordance to your suggestions.

Revisions have been made on page 14 line 269 – 271

• “A higher prevalence of GS was found among individuals with lower socioeconomic status, falling within the first and second terciles of SES.”

This is not totally true when compared to 80+ elderly, widowed, residents in the south and unemployed. Hence, the word ‘higher’ should be reconsidered. Generally, while the reason for not speaking to geographical distribution of GS is best known to the authors, it is important to retain the distribution accordingly: older elderly (80+ years), widowed, southern residence, unemployed, poorest, 70-79 years elderly, females, and northern residence. Afterward, Northeastern residence, average SES, lower education, single, having UCS, having CSMBS, and separated come next in the distribution of GS. By cognitive impairment, older elderly (80+ years), single, as well as widowed and poorest. By functional impairment, older elderly (80+ years), southern residence, and widowed. By frailty, older elderly (80+ years), unemployed, and widowed.

**Response**: We appreciate your constructive comments and suggestions regarding our wording. We have carefully revised the relevant text to clearly mention the high prevalence of geriatric syndrome features in relation to the lower socioeconomic status terciles only.

Our revised text is: “In regard to SES, a higher prevalence of GS was found among individuals with lower socioeconomic status, falling within the first and second terciles of SES,” on page 16, lines 308–309.

• “…of which the observed inequality was relatively larger among those with cognitive impairment.” EVEN MUCH LARGER AMONG FUNCTIONALLY IMPAIRED.

**Response**: Thank you for your comment. In response, we carefully reviewed our conclusion and revised the relevant text for greater clarity. The larger magnitude refers to the concentration index of cognitive impairment (CI) (C = -0.220) compared to the other two phenotypes: functional impairment (C = -0.082) and frailty (C = -0.130). To clarify, we have added the term 'socioeconomic' to emphasize that the greater magnitude of the concentration index reflects a higher level of socioeconomic inequality in CI within our population.

Our revised text is: “Cognitive impairment, functional decline, and frailty were significantly more prevalent among the poor elderly group, with the observed socioeconomic inequality being relatively larger for those with cognitive impairment.” On page 19 line 395.

• “We used age, gender, marital status, education, working status, health insurance, and region of residence, which were investigated in the NHES-5.28” (Aekplakorn W, Chariyalertsak S, Kessomboon P, Assanangkornchai S, Taneepanichskul S, Putwatana P. Prevalence of diabetes and relationship with socioeconomic status in the Thai population: National health examination survey, 2004–2014. J. Diabetes Res. 2018 ;2018:e1654530.) THE REFERENCE DOES NOT SEEM TO MATCH/PROVE THE CLAIM.

**Response**: We thank you for your comment and understand that the reference title and its main analysis focused on diabetes. However, we selected this article as our reference because it contains the clearest English description (as provided by the principal investigator of the project) of the National Health Survey Method, which was used up until the most recent survey, from which our data was drawn. Additionally, the original report, which includes methodological details, is only available in Thai and may not offer as clear an explanation of the survey methodology for an English-speaking audience as the cited reference.

• List of references seem to be unnecessarily too lengthy, and missing doi. should be included.

**Response**: We understand that the number of references should be kept optimal. However, after we trimmed out the references in the list, almost all the references have been kept due to their relevance to the current study. Any retracted references can be tracked by track changes in the manuscript file as well as are detailed as follows. The articles’ available dois have also been added.

1. Removal due to duplication (n = 1)

Ref8: Inouye SK, Studenski S, Tinetti ME, Kuchel GA. Geriatric syndromes: clinical, research and policy implications of a core geriatric concept. J Am Geriatr Soc. 2007;55(5):780-91.

2. Removal due to redundancy (n = 4)

Ref35. Bornæs O, Andersen AL, Houlind MB, Kallemose T, Tavenier J, Aharaz A, et al. Mild cognitive impairment is associated with poorer nutritional status on hospital admission and after discharge in acutely hospitalized older patients. Geriatrics. 2022; 7(5):95.

Ref39.	Kojima G, Iliffe S, Jivraj S, Walters K. Association between frailty and quality of life among community-dwelling older people: A systematic review and meta-analysis. J Epidemiol Community Health. 2016 Jul; 70(7):716-21.

Ref40.	Kojima G, Iliffe S, Walters K. Frailty index as a predictor of mortality: A systematic review and meta-analysis. Age Ageing. 2018 ; 47(2):193-200.

Ref59: Blom J, den Elzen W, van Houwelingen AH, Heijmans M, Stijnen T, Van den Hout W, et al. Effectiveness and cost-effectiveness of a proactive, goal-oriented, integrated care model in general practice for older people. A cluster randomised controlled trial: Integrated Systematic Care for older People—the ISCOPE study. Age and Ageing. 2016; 45(1):30-41.
